# Osteoprotegerin in relation to insulin resistance and blood lipids in sub-Saharan African women with and without abdominal obesity

**DOI:** 10.1186/s13098-015-0042-3

**Published:** 2015-05-23

**Authors:** Clarisse Noël Ayina Ayina, Eugene Sobngwi, Mickael Essouma, Jean Jacques N. Noubiap, Philippe Boudou, Laurent Serge Etoundi Ngoa, Jean François Gautier

**Affiliations:** Department of Animal Science, Faculty of Science, University of Douala, Douala, Cameroon; Department of Internal Medicine and Specialties, Faculty of Medicine and Biomedical Sciences, University of Yaoundé I, Yaoundé, Cameroon; Laboratory for Molecular Medicine and Metabolism, Biotechnology Center, University of Yaoundé I, Yaoundé, Cameroon; National Obesity Center, Yaoundé Central Hospital, Yaoundé, Cameroon; Department of Medicine, Groote Schuur Hospital and University of Cape Town, Cape Town, South Africa; Medical Diagnostic Center, Yaoundé, Cameroon; Department of Hormonal Biology, Saint-Louis Hospital, Public Assistance - Paris Hospitals, University Paris-Diderot Paris-7, Paris, France; Department of Diabetes and Endocrinology, Saint-Louis Hospital, Public Assistance - Paris Hospitals, University Paris-Diderot Paris-7, Paris, France; Department of Animal Science, Higher Teacher’s Training College, University of Yaoundé I, Yaoundé, Cameroon; INSERM UMRS 1138, Cordeliers Research Centre, University Pierre et Marie Curie-Paris 6, Paris, France

**Keywords:** Osteoprotegerin, Insulin resistance, Lipids, Obesity, Sub-Saharan Africans

## Abstract

**Background:**

Osteoprotegerin (OPG), a soluble member of the tumor necrosis factor receptor superfamily that inhibits bone resorption, has been suggested as a potential marker of cardiovascular risk. This study aimed to assess the relationship between insulin resistance, lipid profile and OPG levels in obese and non-obese sub-Saharan African women.

**Methods:**

Sixty obese (44) and non-obese (16) volunteer women aged 18 to 40 years were recruited in this cross-sectional study. Their clinical (age, height, weight, waist circumference, systolic and diastolic blood pressures) and biochemical parameters (fasting blood glucose, total cholesterol, high density lipoprotein-cholesterol (HDL-C)) were measured using standard methods. Insulin levels were measured using an electrochemiluminescence immunoassay, while OPG levels were measured using the ELISA technique. Low density lipoprotein-cholesterol (LDL-C), body mass index (BMI) and Homeostasis Model Assessment of Insulin Resistance (HOMA-IR) were calculated using standard methods. Abdominal obesity was defined as a waist circumference ≥ 80 cm.

**Results:**

OPG levels were higher in obese than in normal subjects, though the difference was not significant (*p* = 0.9). BMI, waist circumference, percent body fat and systolic blood pressure were significantly higher in obese than in non-obese subjects (p < 0.05). In these subjects, only age significantly correlated with OPG levels (*r* = 0.831, *p* = 0.003), while none of the anthropometric nor metabolic parameter did, even after adjustment for age. In obese subjects, OPG levels fairly correlated with HDL-C (*r* = 0.298, *p* = 0.058), and significantly correlated with HOMA-IR (*r* = −0.438, *p* = 0.018). After adjustment for age, OPG levels remained negatively correlated to HOMA-IR (*r* = −0.516, *p* = 0.020) and LDL-C (*r* = −0.535, *p* = 0.015) and positively correlated to HDL-C (*r* = 0.615, *p* = 0.004). In multiple linear regression analysis, age was a main determinant of OPG levels in non-obese (β = 0.647, *p* = 0.006) and obese (β = 0.356, *p* = 0.044) women. HDL-C was also associated to OPG levels in obese women (β = 0.535, *p* = 0.009).

**Conclusion:**

The positive correlation of OPG with HDL-C and HOMA-IR, and its negative correlation with LDL-C suggest that it may be a marker of insulin sensitivity/resistance and atherogenic risk in obese African women.

## Background

Osteoprotegerin (OPG) is a soluble glycoprotein that belongs to the tumor necrosis factor receptor super-family [1]. It acts as a decoy soluble receptor for the receptor activator of nuclear factor kB ligand (RANKL) [2], thus preventing RANKL from binding its receptor on osteoclasts, thereby inhibiting osteoclastogenesis [3]. Some studies showed that OPG may modulate vascular calcification, endothelial dysfunction and left ventricular dysfunction [4–7]. OPG was also found associated to ischemic heart disease, stroke and all-cause mortality in patients with heart failure [4, 8–10]. Furthermore, a relation between OPG and insulin sensitivity was seen in an ageing male population, and in obese and non-obese individuals [11, 12]. There is therefore a need to further investigate the potential of OPG as a marker of cardiovascular risk in diverse ethnic populations. This study aimed to assess the relationship between insulin resistance, lipid profile and OPG levels in obese and non-obese sub-Saharan African women.

## Methods

This was a cross-sectional study. Sixty women aged 18 to 40 years, of which 44 obese and 16 non obese were recruited within the general population of Edéa, Cameroon. For each participant we measured height, waist circumference to the nearest 0.5 cm, and weight in light clothes to the nearest 0.1 kg, and we then calculated the body mass index (BMI) as weight in kg/height^2^ in m^2^. We measured resting blood pressures using standardized procedures with an automatic sphygmomanometer Omron HEM-705 CP (Omron Corporation, Tokyo, Japan). Fasting blood glucose was obtained with the Accu-Chek® Compact Plus glucometer (F. Hoffmann-La Roche AG, Basel, Switzerland). Serum cholesterol (cholesterol oxidase phenol4-amino antipyrene peroxidase method), serum triglycerides (glycerol phosphatase oxidase-phenol4-amino antipyrene peroxidase method), and high-density lipoprotein (HDL)-cholesterol (cholesterol oxidase phenol4-amino antipyrene peroxidase method) were measured on a spectrophotometer (UV Mini 1240) using Chronolab kits (Chronolab Systems, Barcelona, Spain). Low-density lipoprotein (LDL)-cholesterol was calculated using the Friedwald’s formula [13]. Insulin levels were measured using an electrochemiluminescence immunoassay (Roche Diagnostics, Indianapolis, USA), while OPG levels were measured using Osteoprotegerin ELISA kit (Immunodiagnostic systems, Fontain Hill, USA). Homeostasis Model Assessment of Insulin Resistance (HOMA-IR) calculated as HOMA ‐ IR = (*Fasting insulin* × *Fasting blood glucose*) ÷ 22.5. Abdominal obesity was defined as a waist circumference ≥ 80 cm.

Data were coded, entered and analyzed using the Statistical Package for Social Science (SPSS) version 20.0 for Windows (IBM Corp. Released 2011. IBM SPSS Statistics for Windows, Version 20.0. Armonk, NY: IBM Corp.). Variables were expressed as mean with standard deviation (SD). The Student t-test was used to compare the obese and non-obese groups with respect to clinical and biological parameters. Pearson’s correlation and partial correlation were used to determine unadjusted and adjusted correlates of OPG levels in obese women. Multiple stepwise linear regression analyses were performed to determine the metabolic correlates of OPG levels in obese and non-obese women. A *p* value < 0.05 was considered statistically significant.

The study was approved by the National Ethical Review Board of the Cameroon Ministry of Public Health. Written informed consent was obtained from all the participants. The study was conducted in accordance with the Helsinki Declaration.

## Results

Sixty women (44 obese and 16 non-obese) were recruited. Obese women were significantly older than non-obese (mean age: 31.86 ± 5.23 vs 27.44 ± 6.07; *p* = 0.012). BMI, waist circumference, systolic blood pressure and percent body fat levels were significantly higher in obese than in non-obese subjects (p<0.05). OPG levels were comparable among both groups (Table 1).

**Table 1:** General characteristics of the study population

Characteristics	Non obese	Obese	*p*
Age (years)	27.44 (6.07)	31.86 (5.23)	0.012
BMI (kg/m^2^)	22.09 (1.89)	30.35 (5.86)	0.000
Waist circumference (cm)	73.44 (4.29)	96.91 (12.94)	0.000
% body fat	19.76 (5.52)	32.03 (8.68)	0.000
Systolic arterial pression (mmHg)	110 (18)	122 (18)	0.012
Diastolic arterial pression (mmHg)	74 (11)	82 (16)	0.103
Blood glucose (mmol/L)	4.63 (0.63)	5.04 (2.01)	0.933
Triglycerides (mmol/L)	1.21 (0.47)	1.26 (0.50)	0.894
LDL-Cholesterol (mmol/L)	1.84 (0.90)	1.69 (1.06)	0.387
HDL-Cholesterol (mmol/L)	2.23 (0.59)	2.06 (0.83)	0.385
Insulin (UI/ML)	2.25 (0.55)	3.85 (0.65)	0.207
HOMA-IR	9.19 (7.60)	14.50 (12.58)	0.126
Osteoprotegerin (pmol/L)	4.14 (1.84)	4.16 (1.78)	0.900

Data are mean (standard deviation)

BMI: body mass index; % Body fat: percentage of body fat; LDL-Cholesterol: Low density lipoprotein cholesterol; HDL-Cholesterol: High density lipoprotein cholesterol; HOMA-IR: Homeostasis Model assessment of insulin resistance

In non-obese subjects, only age significantly correlated with OPG levels (*r* = 0.831, *p* = 0.003), while none of the anthropometric nor metabolic parameters did, even after adjustment for age. As shown in Table 2, in obese subjects, OPG levels were almost correlated with HDL-C (*r* = 0.298, *p* = 0.058), and were significantly correlated with HOMA-IR (*r* = −0.438, p = 0.018). After adjustment for age, OPG levels remained negatively correlated to HOMA-IR (*r* = −0.516, *p* = 0.020) and LDL-C (*r* = −0.535, *p* = 0.015) and positively correlated to HDL-C (*r* = 0.615, *p* = 0.004).

**Table 2:** Unadjusted and adjusted metabolic correlates of osteoprotegerin in obese women

Correlates	Model 1	Model 2
	Correlation coefficient (r)	
LDL-Cholesterol	−0.057	−0.535*
HDL-Cholesterol	0.298	0.615**
HOMA-IR	−0.438*	−0.516*

Model 1: Unadjusted correlates

Model 2: Adjusted correlates (adjustment for age)

LDL-Cholesterol: Low density lipoprotein cholesterol; HDL-Cholesterol: High density lipoprotein cholesterol; HOMA-IR: Homeostasis model assessment of insulin resistance

*p<0.05; **p<0.01

In multiple stepwise regression analysis (Table 3), age was a main determinant of OPG levels in non-obese (β =0.647, *p* = 0.006) and obese (β =0.356, *p* = 0.044) women. HDL-C was also associated to OPG levels in obese women (β = 0.535, *p* = 0.009).

**Table 3:** Linear regression associating osteoprotegerin levels with age and metabolic parameters

Correlates	Non obese	Obese
	Coefficient (β)	
Age	0.647**	0.356*
HDL-Cholesterol	0.064	0.535**
Triglycerides	−0.429*	−0.019

HDL-Cholesterol: High density lipoprotein cholesterol

*p<0.05; **p<0.01

## Discussion

The aim of this study was to assess the relationship between insulin resistance, lipid profile and OPG levels in obese and non-obese sub-Saharan African women. Age significantly correlated with OPG levels in both groups. After adjustment for age, only HDL-C levels, LDL-C levels, and HOMA-IR correlated with OPG levels in obese, but not non-obese women.

OPG levels have been associated with a high risk of cardiovascular disease in humans [14, 15]. This result has long been a matter of controversy in the literature, possibly because of different sizes of the study populations and of different methodologies [4]. Even in our previous study carried out on an older population made up of both sexes, HDL-C levels did not correlate with OPG levels [16]. Some observational studies showed an association between elevated serum OPG levels and clinical cardiovascular disease [11, 12, 17, 18]. Unlike these studies, we found that HDL-C which has a protective effect on heart and vessels in humans [19], was positively correlated with OPG levels. Additionally, LDL-C which carries a proatherogenic profile was negatively correlated to OPG. These findings are consistent with evidence from many animals studies that found a protective role for OPG on the vasculature [20].

We found that HOMA-IR was negatively correlated with OPG levels in obese women, indicating that elevated OPG levels may be suggestive of high insulin sensitivity. Contrariwise, in a study among Caucasian obese, HOMA-IR was positively correlated to OPG levels [10]. Although our findings along with those of previous studies suggest that serum OPG may be a marker of insulin sensitivity/resistance in obese individuals [10, 12], whether elevated serum OPG levels reflect insulin sensitivity or resistance in this particular population remains controversial. Differences in ethnic background, metabolic conditions and diagnostic criteria of insulin sensitivity using HOMA-IR in the populations studied may partly explain this inconsistency. Indeed, it has been shown that cut-off values of HOMA-IR are defined by population-based percentiles criteria. Furthermore, these cut-off values are different according to ethnicity, clinical methods of estimation, and metabolic conditions of populations studied [21]. To the best of our knowledge, there is no reference values for HOMA-IR that have been validated in our population. However, we conducted a study that showed that the performance of fasting insulin sensitivity indices in predicting insulin sensitivity among Black Africans are not optimal [22]. Overall, discrepancy between our results and some previous reports in Caucasians regarding the relationship between OPG levels and lipid profile and HOMA-IR [10, 12] stresses the need for further investigation with an emphasis on ethnic differences.

The main limitation of this study is its small sample size which hampered definitive inferences and drawing relevant conclusions. Thus, our findings need to be verified in further large population studies.

## Conclusion

The positive correlation of OPG with HDL-C and HOMA-IR, and its negative correlation with LDL-C suggest that it maybe a marker of insulin sensitivity/resistance and atherogenic risk in obese African women. Further large studies are needed to verify these findings.
